# Whole Transcriptome Data Analysis Reveals Prognostic Signature Genes for Overall Survival Prediction in Diffuse Large B Cell Lymphoma

**DOI:** 10.3389/fgene.2021.648800

**Published:** 2021-06-09

**Authors:** Mengmeng Pan, Pingping Yang, Fangce Wang, Xiu Luo, Bing Li, Yi Ding, Huina Lu, Yan Dong, Wenjun Zhang, Bing Xiu, Aibin Liang

**Affiliations:** ^1^Department of Hematology, Tongji Hospital, Tongji University School of Medicine, Shanghai, China; ^2^National Research Center for Translational Medicine at Shanghai, Ruijin Hospital Affiliated to Shanghai Jiao Tong University School of Medicine, Shanghai, China

**Keywords:** diffuse large B cell lymphoma, overall survival, prognosis, biomarkers, risk score

## Abstract

**Background:**

With the improvement of clinical treatment outcomes in diffuse large B cell lymphoma (DLBCL), the high rate of relapse in DLBCL patients is still an established barrier, as the therapeutic strategy selection based on potential targets remains unsatisfactory. Therefore, there is an urgent need in further exploration of prognostic biomarkers so as to improve the prognosis of DLBCL.

**Methods:**

The univariable and multivariable Cox regression models were employed to screen out gene signatures for DLBCL overall survival (OS) prediction. The differential expression analysis was used to identify representative genes in high-risk and low-risk groups, respectively, where student *t* test and fold change were implemented. The functional difference between the high-risk and low-risk groups was identified by the gene set enrichment analysis.

**Results:**

We conducted a systematic data analysis to screen the candidate genes significantly associated with OS of DLBCL in three NCBI Gene Expression Omnibus (GEO) datasets. To construct a prognostic model, five genes (*CEBPA*, *CYP27A1*, *LST1*, *MREG*, and *TARP*) were then screened and tested using the multivariable Cox model and the stepwise regression method. Kaplan–Meier curve confirmed the good predictive performance of this five-gene Cox model. Thereafter, the prognostic model and the expression levels of the five genes were validated by means of an independent dataset. High expression levels of these five genes were significantly associated with favorable prognosis in DLBCL, both in training and validation datasets. Additionally, further analysis revealed the independent value and superiority of this prognostic model in risk prediction. Functional enrichment analysis revealed some vital pathways responsible for unfavorable outcome and potential therapeutic targets in DLBCL.

**Conclusion:**

We developed a five-gene Cox model for the clinical outcome prediction of DLBCL patients. Meanwhile, potential drug selection using this model can help clinicians to improve the clinical practice for the benefit of patients.

## Introduction

Diffuse large B cell lymphoma (DLBCL) is the most common type of aggressive non-Hodgkin lymphoma with an annual incidence of 1–5/10,000 (Li et al., 2018; Marangon et al., 2019). DLBCL is an aggressive and potentially curable hematological malignancy, which makes an early diagnosis and effective treatments essential for patients. R-CHOP (rituximab, cyclophosphamide, doxorubicin, vincristine, and prednisone) is currently the standard first line treatment of DLBCL (Coiffier et al., 2002). Despite the high rate of complete response (76%), approximately 40% of patients will relapse, and the molecular mechanism underlying recurrence remains largely unknown (Coiffier et al., 2010). DLBCL displays tremendous clinical, genetic and molecular heterogeneity. The International Prognostic Index (IPI) has been used to predict the prognosis of patients with DLBCL for nearly 30 years, yet there still exists a minority of patients whose clinical process were not in accord with the IPI stratification (International Non-Hodgkin’s Lymphoma Prognostic Factors Project, 1993). Gene expression profiling has helped identify two major subtypes, known as germinal center B-cell-like (GCB) and activated B-cell-like (ABC), and patients with ABC DLBCL exhibit a generally worse prognosis (Lenz et al., 2008a). However, the high prices and strict requirements regarding tissue limit the routine use of this method. Therefore, efforts have been made to find novel biomarkers with prognostic values in order to improve therapeutic strategy selection based on potential targets (Cabanillas and Shah, 2017).

Currently, various markers are defined through immunophenotyping, such as CD5, CD30, BCL2, MYC, and TP53 (Pierce and Mehta, 2017; Zhao et al., 2019). CD5 promotes downstream B-cell receptor signaling, is associated with ABC subtype and more aggressive clinical traits. Patients with CD30^+^ DLBCL, which leads to the downregulation of NF-κB and B-cell receptor signaling, tend to exhibit a better prognosis (Bhatt et al., 2016; Thakral et al., 2017). Meanwhile, in patients with the GCB subtype, BCL2 and MYC rearrangements would lead to worse prognosis (Visco et al., 2013). TP53 mutation also adversely affects patients’ prognosis (Xu-Monette et al., 2012). Based on the new integrated genetic map, Chapuy et al. (2018) identified distinct subsets, including a previously unrecognized group of low-risk ABC-DLBCLs, two GCB-DLBCLs subsets with different prognoses and an ABC/GCB-independent group. In addition, Schmitz et al. (2018) uncovered some previously unknown subtypes of DLBCL by differences in gene-expression signatures and responses to immunochemotherapy. The subset of high-risk patients requires revolutionized therapeutics, and personalized therapy based on patient’s histological and molecular-genetic characteristics will bring greater benefits to patients. Therefore, further exploration of prognostic indicators is still needed to distinguish DLBCL patients of varied prognosis.

## Materials and Methods

### Data Collection

The gene expression data and corresponding clinical information were collected from NCBI Gene Expression Omnibus (GEO) database with accession numbers of GSE32918 (Barrans et al., 2012) (*n* = 172), GSE4475 (Hummel et al., 2006) (*n* = 166), GSE69051 (Sha et al., 2015) (*n* = 149), TCGA (Schmitz et al., 2018) (*n* = 43), GSE31312 (Visco et al., 2012) (*n* = 470), GSE34171 (Monti et al., 2012) (*n* = 68), GSE11318 (Lenz et al., 2008b) (*n* = 203), and GSE10846 (Lenz et al., 2008a) (*n* = 414). It should be noted that Burkitt lymphoma samples in GSE69051 and GSE4475 have been excluded in this study. Among these datasets, GSE32918, GSE4475, and GSE69051 were used for feature selection and model training, while the remaining datasets including TCGA, GSE31312, GSE34171, GSE11318, and GSE10846 were used as independent validation datasets. The expression values were normalized by the data submitters, and discretized by median values, which were used for downstream analysis.

### Cox Proportional Hazard Model

The univariable Cox proportional hazard model was used to screen prognostic genes in the first three datasets. To integrate the three datasets and remove batch effect, we converted the continuous expression values of the shared genes into two discrete expression levels, i.e., high and low expression, using the median expression as the threshold value. The principal component analysis based on the discretized expression levels revealed that no clear batch effect was observed between the three datasets (Kruskal–Wallis test for the top two principal components, *P*-value > 0.05, Supplementary Figure 1), suggesting that there was no significant transcriptional difference between the three datasets. The comparison of the clinical factors indicated that there were significant differences in age and proportion of deceased cases among the three datasets (Supplementary Table 1). Those three discretized datasets of the shared prognostic signatures were then merged and used as the training set for the multivariable Cox model, and the stepwise regression method was used to determine the best model based on the Akaike Information Criterion (AIC). The risk scores for the samples of training and validation sets were estimated using the multivariable Cox model based on the expression levels of those five genes. The high- and low-risk groups were stratified based on the median of the risk scores in the training set. The independent value of this risk stratification was also assessed by multivariable Cox model.

### Differential Gene Expression Analysis

The differential gene expression analysis was conducted to identify the genes that were upregulated or downregulated between specific risk groups. The Wilcoxon rank-sum test and fold change methods were employed, and the thresholds of adjusted *p*-value and log2-fold change were determined at 0.05 and 0.5.

### The Pathway Enrichment Analysis

The upregulated genes in each risk group were further investigated using the Kyoto Encyclopedia of Genes and Genomes (KEGG) pathway enrichment analysis, respectively. Hypergeometric test was applied to test the statistical significance of those identified pathways. The threshold for adjusted *P*-value was determined at 0.05.

### The Drug-Target Identification

The therapeutic targets were selected from the upregulated genes in each risk group. The drugs and upregulated genes were mapped by the R package maftools with *drugInteractions*.

## Results

### Systematic Identification of Prognostic Gene Signatures for Overall Survival Prediction

**FIGURE 1:**
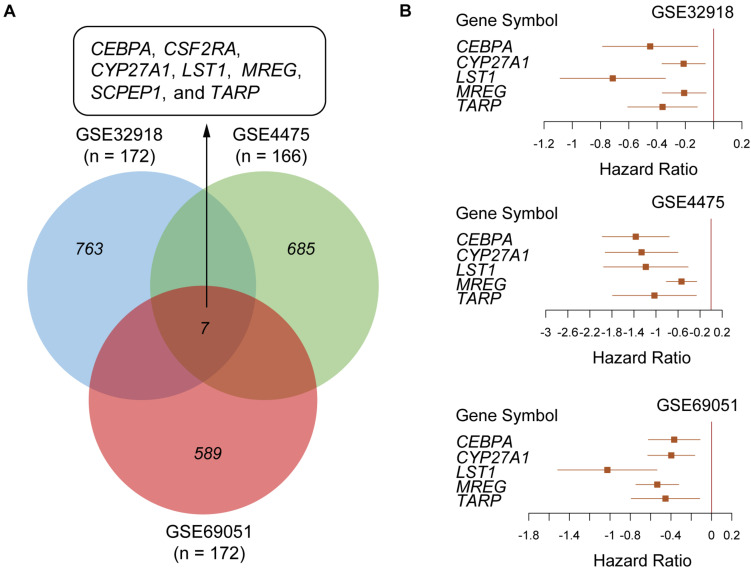
Screening a five-gene Cox model in the public DLBCL datasets from Gene Expression Omnibus (GEO). **(A)** Venn diagram summarizing the overlap between the prognostic genes identified by univariable Cox regression analysis in three public DLBCL datasets with accession numbers of GSE32918 (*n* = 172), GSE4475 (*n* = 166) and GSE69051 (*n* = 172). **(B)** The forest plots represent the association of the five gene signatures with overall survival in the three public DLBCL datasets.

To identify the prognostic gene signatures, we collected three public DLBCL datasets with accession numbers of GSE32918 (*n* = 172), GSE4475 (*n* = 166), and GSE69051 (*n* = 149) from GEO database as depicted in the flow chart in Supplementary Figure 1. Subsequently, univariable Cox regression analysis was conducted, and a total of 763, 685, and 589 genes were identified to be associated with overall survival (OS) based on the gene expression profiles of these three datasets (Figure 1A, log-rank test, *P* < 0.01), respectively. Particularly, *CEBPA*, *CSF2RA*, *CYP27A1*, *LST1*, *MREG*, *SCPEP1*, and *TARP* were found to be significantly associated with OS in all the three datasets at the stringent threshold (Figure 1A). Furthermore, the three datasets were merged into one training set (*n* = 487), and a multivariable Cox regression model was then built from gene expression profiles of the merged dataset. A stepwise method was used to select a subset of those gene signatures to construct a multivariable Cox regression model that could achieve the highest performance. Specifically, five genes including *CEBPA*, *CYP27A1*, *LST1*, *MREG*, and *TARP* were retained in the multivariable Cox model (Table 1), which was termed as the five-gene Cox model, and all of them were associated with favorable prognoses (Figure 1B).

**TABLE 1:** The statistics for the gene signatures in the multivariable Cox model.

**Gene**	**coef**	**exp (coef)**	**se(coef)**	***Z***	**Pr(> | z|)**
*CEBPA*	−0.384	0.681	0.180	−2.138	3.25E-02
*CYP27A1*	−0.390	0.677	0.187	−2.086	3.69E-02
*LST1*	−0.468	0.626	0.178	−2.631	8.50E-03
*MREG*	−0.420	0.657	0.170	−2.471	1.35E-02
*TARP*	−0.292	0.746	0.156	−1.873	6.11E-02

### Performance Validation in an Independent Dataset

**FIGURE 2:**
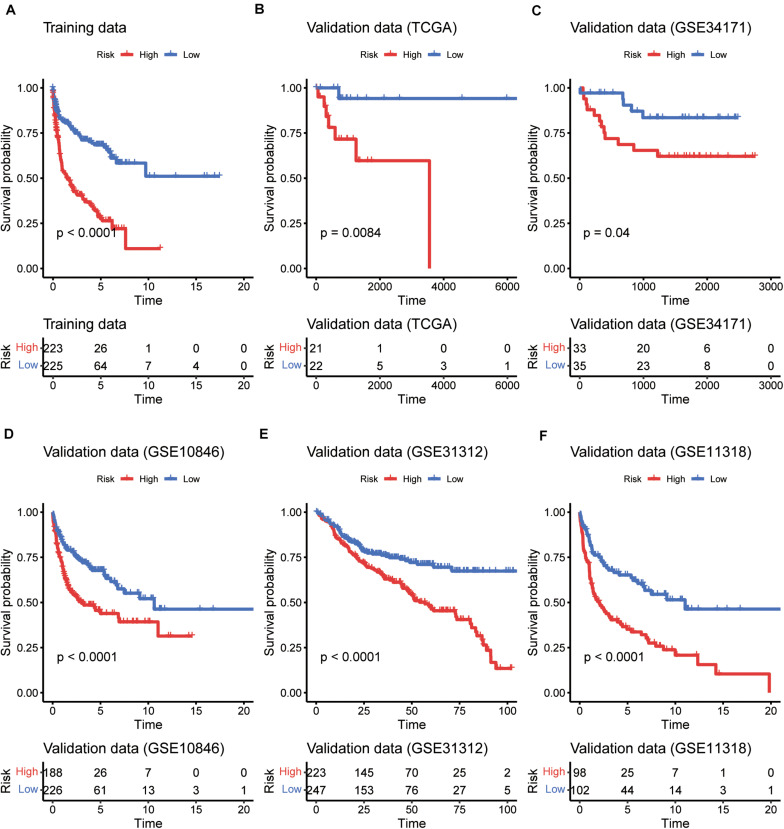
The performance of the five gene signatures in predicting the patients’ risk. K-M curves for the prognostic model in the training datasets **(A)** and the five validation datasets **(B–F)**. The red and blue lines represent the high- and low-risk groups, respectively. The numbers within risk tables on the bottom represent the number of survivors at that time point.

**FIGURE 3:**
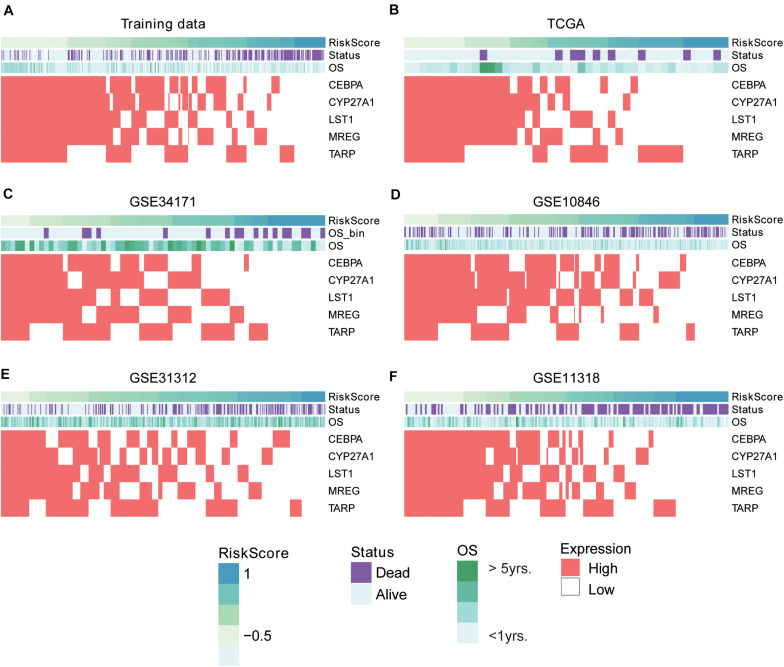
The expression patterns of five prognostic gene signatures in the training and five validation sets. The expression patterns of the five prognostic genes in training **(A)** and validation **(B–F)** sets. The risk scores were estimated by the linear predictors of the Cox model. The samples were ordered by the risk scores.

To evaluate the performance of the multivariable model in risk prediction, we first calculated the risk scores of the DLBCL samples in the training set, and stratified these samples into high- and low-risk groups by the median of risk scores. The high-risk group exhibited worse prognosis than the low-risk group (Figure 2A, *P* < 0.0001). Moreover, we also collected five independent gene expression datasets with long-term follow-up (TCGA, GSE31312, GSE34171, GSE11318, and GSE10846), predicted the risk scores and stratified the samples of those datasets into high- and low-risk groups. Consistently, these two groups also had significant difference in prognosis (Figures 2B–F, *P* < 0.05). Furthermore, the five gene signatures were found to be upregulated in low-risk group than high-risk group in both the training (Figure 3A) and validation sets (Figures 3B–F). These results indicated that these five gene signatures were robust and consistently associated with OS in both training and validation datasets.

### The Five-Gene Cox Model Is Superior to Other Gene Expression-Based Cox Models

**FIGURE 4:**
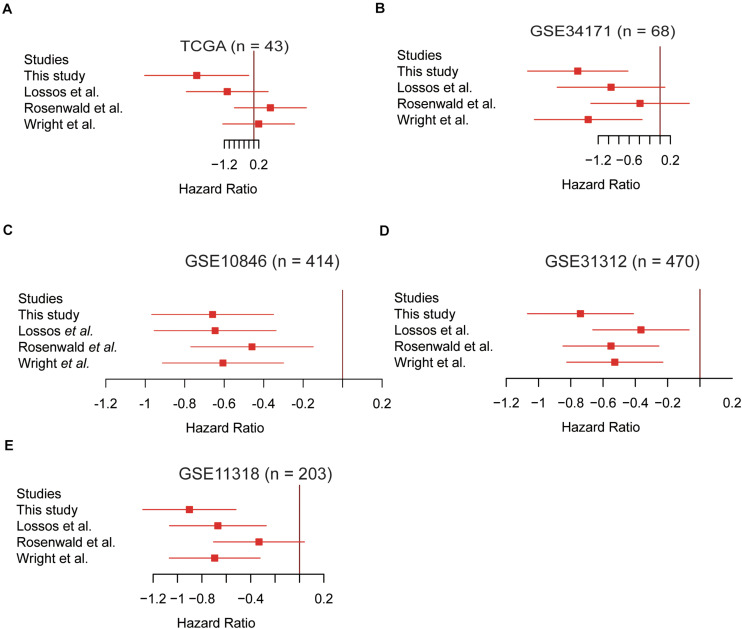
The Cox model based on the five gene signatures was superior to other models. The performance of the four prognostic models in the validation datasets of TCGA (*n* = 43), GSE34171 (*n* = 68), GSE10846 (*n* = 414), GSE31312 (*n* = 470), and GSE11318 (*n* = 203) are displayed in panels **(A–E)**. The log2-hazard ratios and 95% confidence intervals were denoted by the red boxes and lines.

To demonstrate the superiority of this five-gene Cox model based on the five gene signatures, we compared its performance with three sets of gene signatures (Rosenwald et al., 2002; Wright et al., 2003; Lossos, 2008) on the five validation datasets. Utilizing the trained models that were constructed from different gene signatures, the samples in the validation sets could also be stratified into high- and low-risk groups. The gene signatures proposed by Rosenwald et al. (2002) had the worst performance on almost all validation datasets (Figure 4). However, survival difference between samples stratified by our proposed five gene signatures was the most statistically significant across all the validation datasets (Figure 4), especially in the TCGA and GSE34171 cohorts with smaller sample size (Figures 4A,B), suggesting that the Cox model based on our five gene signatures was superior to other models.

### The Five-Gene-Based Risk Stratification Is a Prognostic Factor Independent of Clinical Factors

**FIGURE 5:**
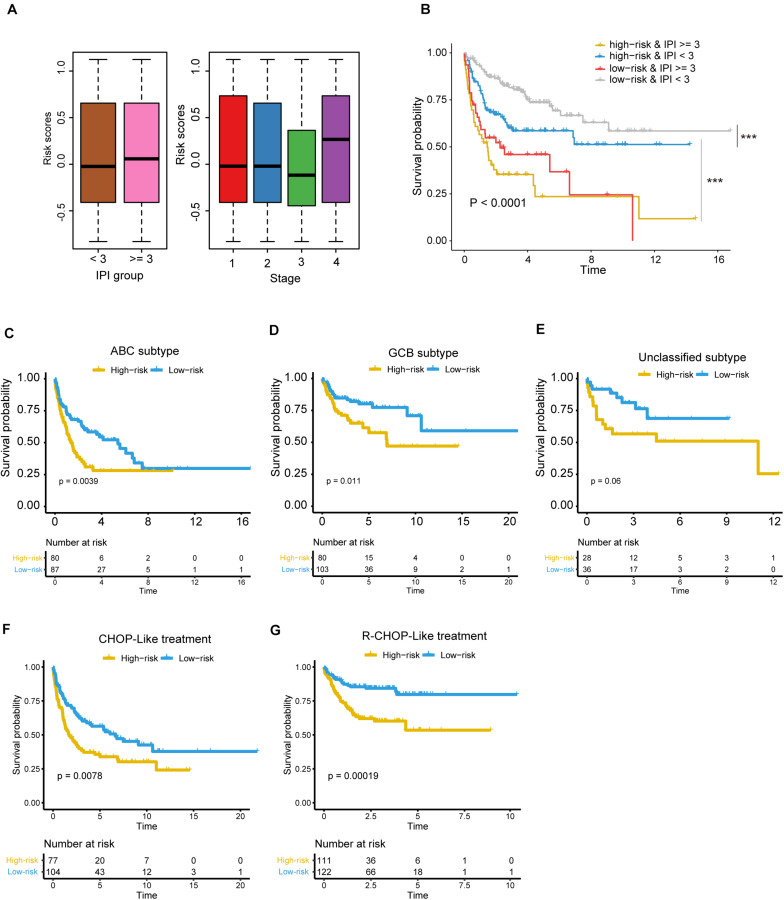
The risk stratification based on the five prognostic genes is independent of clinical factors. **(A)** The risk scores in different IPI groups (left panel) and clinical stages (right panel). The boxes show the median and the interquartile range (IQR) of the risk scores grouped by the IPI scoring system and clinical stage in the validation dataset. There are no significant differences between those groups (*P* > 0.05). **(B)** Kaplan–Meier survival curves show the overall survival of samples grouped by combining the IPI scoring system and the five-gene-based risk stratification. ***P < 0.0001. The differences of overall survival between the high-risk and low-risk groups in specific subtype or with specific chemotherapy regiment [**(C)** ABC subtype; **(D)** GCB subtype; **(E)** unclassified subtype, **(F)** DLBCL treated with CHOP-Like regiment, **(G)** DLBCL treated with R-CHOP-Like regiment].

To further investigate the robustness of the five-gene Cox model, we tested whether the five-gene-based risk stratification was an independent predictor in the validation set. Since the IPI scoring system was a well-recognized factor for prognostic risk prediction and widely applied in clinical practice (Martelli et al., 2013), the samples were first divided into two groups of high (≥3) or low (<3) IPI scores, considering age, serum lactate dehydrogenase (LDH), Eastern Cooperative Oncology Group (ECOG) Performance Status, Ann Arbor stage, and extranodal infiltration sites (International Non-Hodgkin’s Lymphoma Prognostic Factors Project, 1993). As shown in Figure 5A, no significant difference was observed between the risk scores of the two groups, which were estimated using the five-gene Cox model (high vs. low IPI). Moreover, the differences were also not observed across the four stages. In contrast, the samples with high IPI had significantly higher risk scores when estimated with the three sets of gene signatures as mentioned above, than those with low IPI (Supplementary Figure 2). These results suggested that the risk scores were not only irrelevant to IPI scoring system and tumor stage, but also had a higher independent predictive values than those derived from previous gene signatures.

Notably, the samples could be classified into four groups by combining the IPI scoring system and the five-gene-based risk stratification, and the four groups exhibited significantly prognostic difference (Figure 5B, *P* < 0.0001). It should be noted that the differences of OS were not observed between the two groups with the worse prognosis, but the samples with IPI ≥ 3 in high-risk group still had shorter OS than samples with IPI ≥ 3 in the low-risk group based on the KM curve.

Moreover, we also tested whether the risk stratification was independent of the DLBCL subtypes. Consistently, the three subtypes, including ABC, GCB and unclassified subtypes, could be further stratified into high- and low-risk groups. Except unclassified subtype, the ABC and GBC subtypes still maintained the statistical difference in OS between the high-risk and low-risk groups (Figures 5C,D, FDR < 0.05, and Figure 5E, FDR > 0.05). To test whether the chemotherapy treatment affects the performance of the gene signatures, we compared the two risk groups of patients treated with R-CHOP-like or CHOP-like regimens. Consistently, high-risk patients, who were treated with R-CHOP-like or CHOP-like regimens, still had shorter OS than the corresponding low-risk patients (Figures 5F,G), suggesting that the gene signatures were independent of the chemotherapy treatment. In addition, we also fitted the IPI scoring system, stage, subtype and risk stratification into a multivariable Cox model, and found that the risk stratification was still statistically significant with these prognostic factors as cofactors (Table 2). These results further demonstrated that the five-gene-based risk stratification was an independent prognostic factor for DLBCL risk prediction.

**TABLE 2:** The statistics for the risk stratification and prognostically clinical factors in the multivariable Cox model.

**Variables**	**Log2 hazard ratio**	**Hazard ratio**	**Standard error**	***Z* score**	***P*-value**
**Subtype**					
ABC					
GCB	−0.94	0.39	0.20	−4.66	3.18E-06
Unclassified	−0.79	0.45	0.27	−2.94	3.26E-03
**Stage**					
1					
2	0.99	2.70	0.41	2.41	1.62E-02
3	0.64	1.89	0.44	1.45	1.47E-01
4	0.99	2.69	0.42	2.34	1.94E-02
**Risk stratification**					
High-risk					
Low-risk	−0.59	0.55	0.18	−3.34	8.46E-04
**IPI**					
<3					
≥3	1.02	2.77	0.21	4.83	1.40E-06
**Treatment**					
R-CHOP					
R-CHOP-like	−0.72	0.48	0.19	−3.74	1.82E-04

### The Molecular Characteristics and Potential Drugs for the Two Risk Groups

**FIGURE 6:**
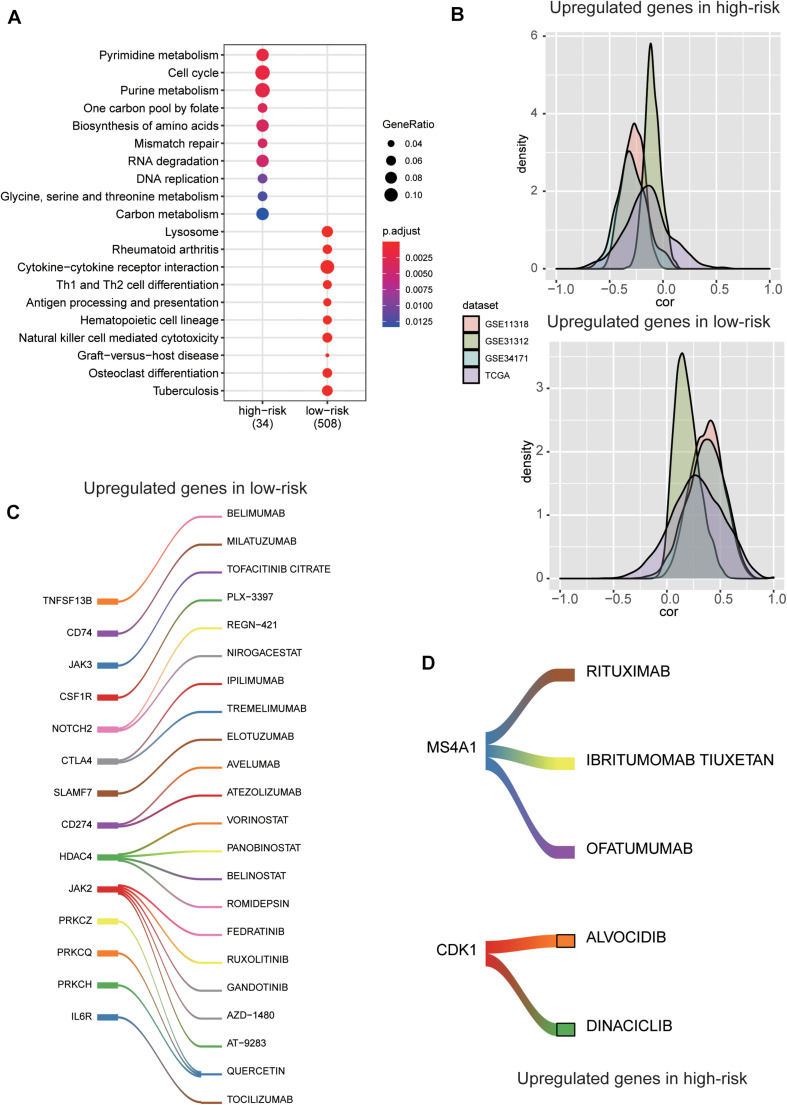
The molecular characteristics and potential drugs for the two risk groups. **(A)** The top-ten GO terms enriched by the upregulated genes in high-risk and low-risk groups. The dots size and color represent the ratio of gene counts and statistical significance, respectively. **(B)** The probability density function of the Spearman’s correlation between the five prognostic genes and the differentially expressed genes (DEGs). The colors represent the validation datasets. **(C)** The upregulated immune checkpoint proteins and the corresponding drugs in the low-risk group. **(D)** The upregulated cell cycle kinase and their potential drugs in high-risk group.

To reveal the molecular characteristics of the two risk groups, we compared the gene expression profiles of high-risk with those of low-risk group using the five validation datasets. A total of 1,158 genes, jointly differentially expressed between high- and low-risk groups of the five validation datasets, were then selected by Wilcoxon rank-sum test and fold change (Adjusted *P*-value < 0.05 and log2-fold change > 0.5). Moreover, the overrepresentation enrichment analysis (ORA) was employed to identify the pathways potentially involved in the DLBCL progression (Figure 6A). Specifically, cell cycle-related pathway and those associated with genomic stability maintenance, such as mismatch repair, were highly upregulated in high-risk group (Adjusted *P*-value < 0.05). In contrast, immune-related pathways such as rheumatoid arthritis, antigen processing and presentation, hematopoietic cell lineage, and Th1 and Th2 cell differentiation were upregulated in low-risk group (Adjusted *P*-value < 0.05). Moreover, we also conducted correlation analysis between our signature genes and the DEGs in the five validation datasets. As high expression of the five signature genes indicates better prognosis, consistently, they are positively or conversely correlated with most of the upregulated genes in high-risk or low groups, respectively, indicating that those DEGs might also be associated with prognosis to a certain extent (Figure 6B).

For the low-risk group, some immune checkpoint proteins and inhibitors were identified, such as PDCD1 (PD-1), CD274 (PD-L1), CTLA4, and their corresponding drugs (Figure 6C), suggesting that the low-risk samples might benefit from inhibiting the immune checkpoint pathway. Besides, the cell cycle kinase, CDK1, was upregulated in high-risk group, and BARASERTIB and DINACICLIB might be the potential drugs for treating DLBCL classified as high-risk (Figure 6D). As we have known, CD20 (also termed *MS4A1*) is expressed on the surface of normal B lymphocytes and is detected in almost all DLBCL cases. At present, RITUXIMAB, a chimeric monoclonal antibody directed against the CD20, combined with intensive chemotherapy (CHOP) is the standard therapy for DLBCL (Figure 6D). These results indicated the stratification may contribute to the selection of targeted drugs for the DLBCL patients.

## Discussion

Diffuse large B cell lymphoma is a remarkably heterogeneous disease, both histologically and genetically. Despite significant advances in subtype classification of DLBCL, accurate prediction of prognosis remains a challenge. With the development of high throughput sequencing technology, some potential prognostic genomic markers for DLBCL patients have been identified (Rosenwald et al., 2002; Wright et al., 2003; Lossos, 2008). However, the number of prognostic markers is still limited. There is an urgent need to screen out more biomarkers to improve the accuracy of prognostic prediction.

In the present study, we identified potential gene candidates through the univariable Cox regression analysis to examine associations between gene expression and patient prognosis of three DLBCL cohorts in GEO. To further narrow down the list of candidate gene signatures, multivariate Cox analysis was carried out on the merged datasets. A stepwise approach was used to select a subset of gene candidates to achieve the highest performance, and a risk model was established for predicting DLBCL prognosis based on the expression levels of five genes including *CEBPA*, *CYP27A1*, *LST1*, *MREG*, and *TARP*. We evaluated the model performance using an independent gene expression dataset and compared it with previously reported models. Our five-gene based risk model showed improved robustness, accuracy, and efficiency compared to those models and was demonstrated to be an independent prognostic factor for OS in patients with DLBCL. Subsequently, we compared the gene expression profiles of high-risk with those of low-risk group and performed ORA to identify pathways potentially involved in the DLBCL progression. Thus, we believe that our five-gene-based risk scoring model can be used for refining DLBCL subtypes and potentially improving patient therapy.

According to the multivariable Cox model, high expression of the five genes was all associated with a favorable survival outcome. CEBPA is a transcription factor playing roles in regulating proliferation and differentiation of many cell types (Gery et al., 2005). Within the hematopoietic system, inactivation mutation of CEBPA blocks the granulocytic differentiation in acute myeloid leukemia (AML) (Wang et al., 1999). In addition, it has been reported that CEBPA-regulated PER2 activation is a potential tumor suppressor pathway in diffuse large B-cell lymphoma (DLBCL) (Thoennissen et al., 2012). CYP27A1, a cytochrome P450 oxidase family member, is closely related to the proliferation of multiple tumor cells, such as prostate, breast and colon cancer (Ji et al., 2016; Alfaqih et al., 2017; Kimbung et al., 2017). LST1 is encoded within the TNF region of the human MHC which regulates lymphocyte proliferation (Rollinger-Holzinger et al., 2000). MREG is reported to suppress thyroid cancer cell invasion and proliferation through PI3K/Akt-mTOR signaling pathway (Meng et al., 2017). The biological roles of these genes in DLBCL need to be further investigated.

The ORA of DEGs suggests that the abnormal cell cycle progression and increased genomic instability contribute to the rapid progression of DLBCL. Inhibitors of cell cycle kinase, such as BARASERTIB and DINACICLIB, may be effective in high-risk patients. On the contrary, genes related to immune-related pathways, such as antigen processing and presentation, Th1 and Th2 cell differentiation, were enriched in low-risk group, suggesting that activated host immune response may indicate favorable prognosis and response to therapy. These findings provide novel clues into the explanation of the mechanisms of DLBCL.

The prognostic model we proposed is helpful for further risk stratification at the genetic level on the basis of the present traditional subtyping, but this study still has some limitations. Some potential prognostic factors may be excluded in the model such as the racial factors and the roles that the five genes play in DLBCL requires further experimental validation. To sum up, our research indicates that the five-gene prognostic model is a reliable tool for predicting the OS of DLBCL patients and providing some hints on drug selection, which can assist clinicians in selecting personalized treatment, although specific drug selection requires further molecular biology research and clinical trials.

## Data Availability Statement

The original contributions presented in the study are included in the article/Supplementary Material, further inquiries can be directed to the corresponding author/s.

## Ethics Statement

Participants gave their written informed consent for the materials to appear in publications without limit on the duration of publication.

## Author Contributions

BX, WZ, and AL conceived and designed the experiments. MP, PY, and FW acquired data, related materials, and analysis tools. MP, XL, and BL analyzed the data. MP, PY, and FW wrote the manuscript. YDi, HL, and YDo revised the manuscript. All authors read and approved the final manuscript.

## Conflict of Interest

The authors declare that the research was conducted in the absence of any commercial or financial relationships that could be construed as a potential conflict of interest.

## Supplementary Material

Supplementary Figure 1 The principal component analysis (PCA) of the discretized expression profiles of the three cohorts used for model training.

Supplementary Figure 2 The association of risk scores derived from the three previous gene signature sets with IPI scoring system and tumor stage.

Supplementary Table 1 Clinical difference between the three cohorts used for model training.

## Abbreviations

DLBCL: diffuse large B cell lymphoma
IPI: International Prognostic Index
GCB: germinal center B-cell -like
ABC: activated B-cell -like
GEO: Gene Expression Omnibus
LDH: serum lactate dehydrogenase
ECOG: Eastern Cooperative Oncology Group
CHOP: combine with intensive chemotherapy
circRNA: circular RNAs
HCC: hepatocellular carcinoma
ncRNA: non-coding RNAs
PVTT: portal vein tumor thrombosis
GSEA: gene set enrichment analysis.
