# Supplementary figures and images for: Whole Transcriptome Data Analysis Reveals Prognostic Signature Genes for Overall Survival Prediction in Diffuse Large B Cell Lymphoma
Source: Front Genet. 2021 Jun 9;12:648800. doi: 10.3389/fgene.2021.648800 (PMC8220154; doi:10.3389/fgene.2021.648800)

# Individuals – PCA

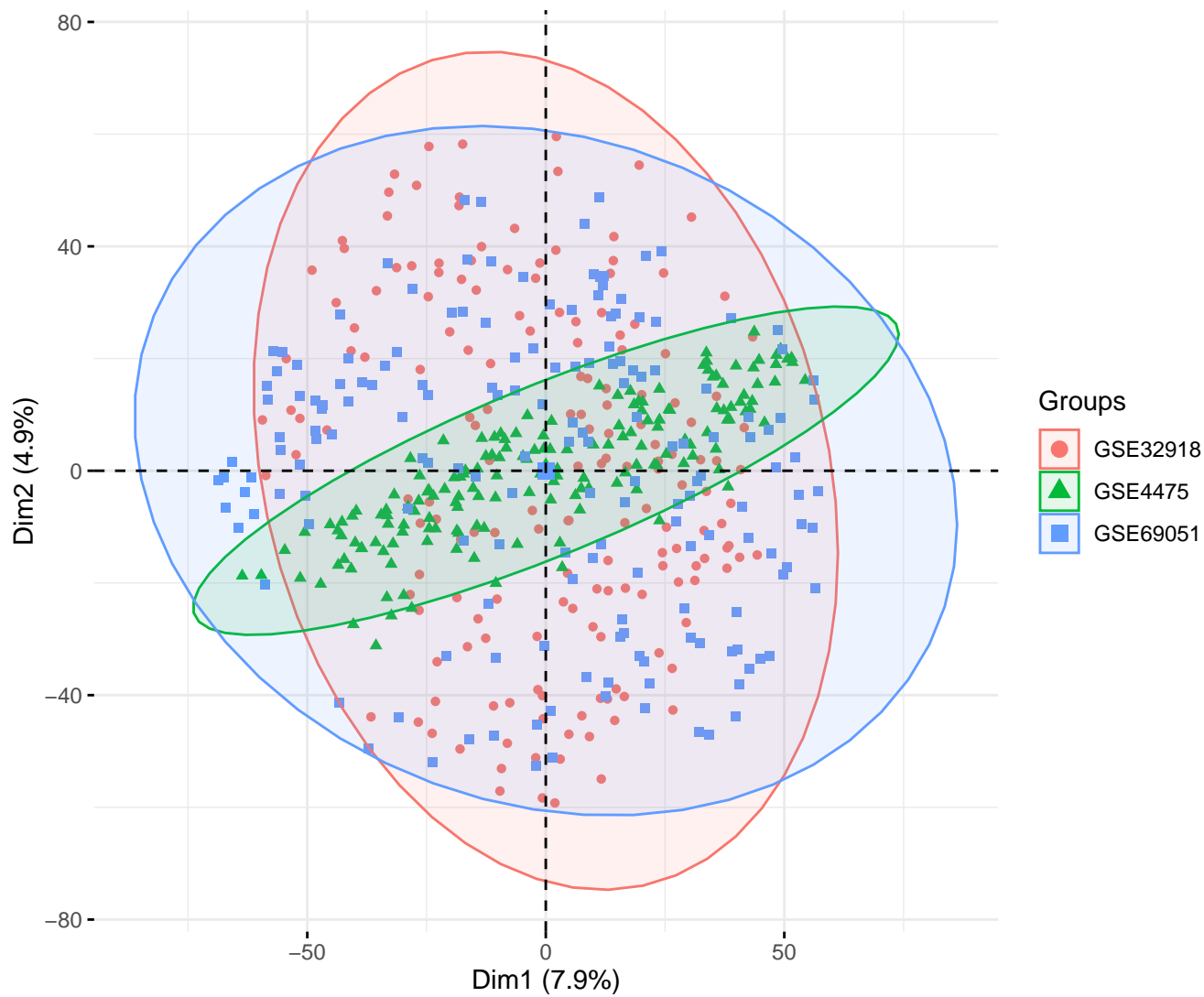

Supplement: Supplementary Figure 1 — The principal component analysis (PCA) of the discretized expression profiles of the three cohorts used for model training. [file Data_Sheet_1.PDF]

A

Lossos et al.

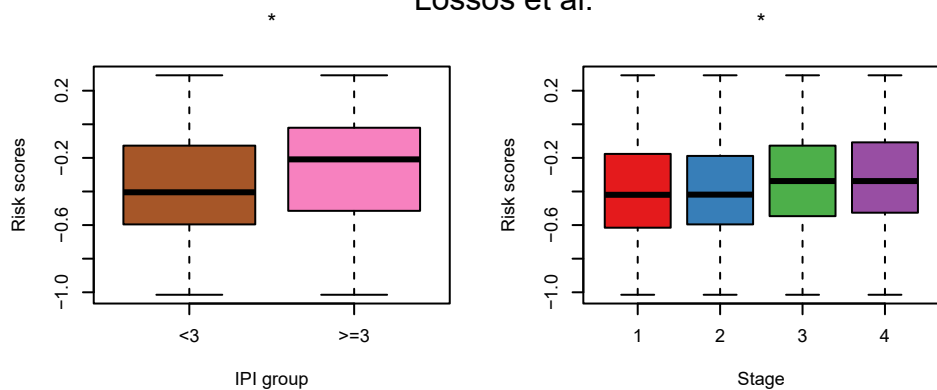

B

Rosenwald et al.

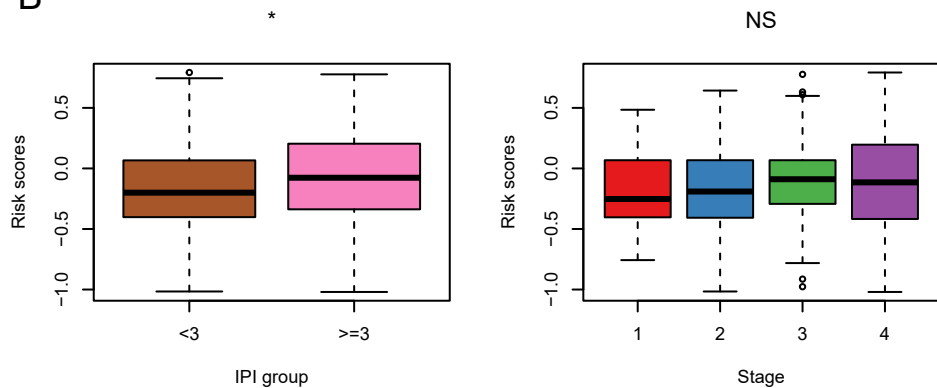

C

Wright et al.

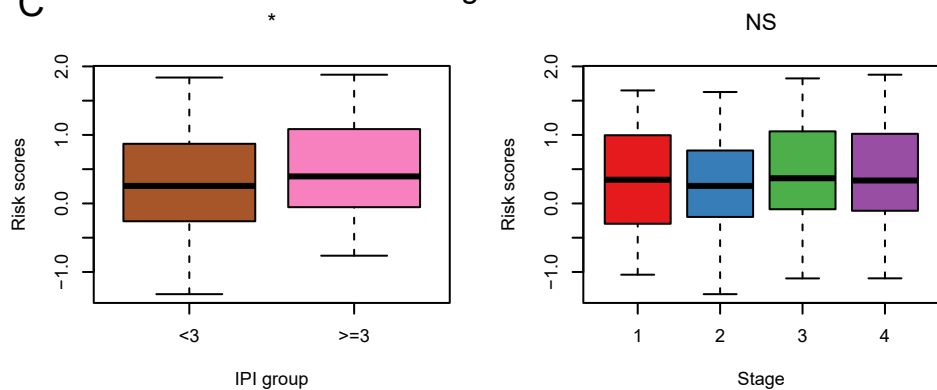

Supplement: Supplementary Figure 2 — The association of risk scores derived from the three previous gene signature sets with IPI scoring system and tumor stage. [file Data_Sheet_2.PDF]
